# Spanish adaptation and validation of the supportive & palliative care indicators tool – SPICT-ES^TM^

**DOI:** 10.11606/S1518-8787.2018052000398

**Published:** 2018-01-16

**Authors:** Alfonso Alonso Fachado, Noemí Sansó Martínez, Marisa Martín Roselló, José Javier Ventosa Rial, Enric Benito Oliver, Rafael Gómez García, José Manuel Fernández García

**Affiliations:** I Servizo Gallego de Saúde. Subdirección General de Planificación y Programacion Asistencial. Santiago de Compostela, Galicia, España; II Universidad de las Islas Baleares. Facultad de Enfermería y Fisioterapia. Mallorca, Islas Baleares, España; III Fundación Cudeca. Málaga, Andalucía, España; IV Complejo Hospitalario Universitario de Ferrol. Servizo Galego de Saúde. Ferrol, Galicia, España; V Universidad Francisco de Vitoria. Madrid, España; VI Centro de Salud de Porto do Son. Servizo Gallego de Saúde. Porto do Son, Galicia, España

**Keywords:** Palliative Care Clinical Decision-Making, Decision Support Techniques, Surveys and Questionnaires, utilization, Translations, Validation Studies, Multicenter Study, Cuidados Paliativos Toma de Decisiones Clínicas, Técnicas de Apoyo para la Decisión, Encuestas y Cuestionarios, utilización, Traducciones, Estudios de Validación, Estudio Multicéntrico

## Abstract

**OBJECTIVE:**

To culturally adapt and validate the SPICT^TM^ to Spanish, which is a brief and simple tool to support a better identification of chronic patients who have palliative care needs.

**METHODS:**

For this study, we designed a multicenter and national project between the centers of Galicia, Balearic Islands, and Andalusia. For the process of translation and cross-cultural adaptation of the SPICT^TM^ to Spanish, we followed the steps proposed by Beaton et al. with successive translations and subsequent consensus of experts using the debriefing methodology. After the content validation was completed, the psychometric properties were validated. A prospective longitudinal study was designed with 188 patients from Galicia, the Balearic Islands, and Andalusia. The internal consistency and reliability of the test and retest was analyzed for 10 days by the same researcher.

**RESULTS:**

For more than 90% of the participants of the SPICT-ES^TM^, it seems simple to be filled out, and they consider it written in an understandable language. The average time to apply the questionnaire without prior knowledge was 4 minutes and 45 seconds. To evaluate the internal consistency of the instrument, we used the Kuder-Richardson formula 20. Internal consistency is 0.71. The agreement index of the Kappa test is between 0.983 and 0.797 for the different items.

**CONCLUSIONS:**

In this study, we demonstrate the equivalence of content with the original. In addition, the validation of the psychometric properties establishes that the SPICT-ES^TM^ maintains adequate reliability and stability. If we add the satisfaction shown by the professionals and the ease of use, the SPICT-ES^TM^ is an adequate tool for the identification of palliative patients with chronic diseases and palliative care needs.

## RESUMEN

**OBJETIVO:**

Adaptar culturalmente al español y validar el SPICT^TM^, una herramienta breve y sencilla para apoyar una mejor identificación de pacientes crónicos que presentan necesidades de atención paliativa.

**MÉTODOS:**

Para este estudio se diseñó un proyecto multicéntrico y nacional entre centros de Galicia, Islas Baleares y Andalucía. Para el proceso de traducción y adaptación transcultural del SPICT^TM^ al castellano, se siguieron los pasos propuestos por Beaton et al. con sucesivas traducciones y posterior consenso de expertos mediante la metodología *debriefing*. Tras finalizar la validación de contenido se procedió a la validación de las propiedades psicométricas. Se diseñó un estudio longitudinal prospectivo en 188 pacientes de Galicia, Baleares y Andalucía. Fue analizada la consistencia interna y la fiabilidad test-retest a los 10 días por el mismo investigador.

**RESULTADOS:**

A más del 90% de los participantes SPICT-ES^TM^ les parece sencillo cumplimentarlo, y consideran que está escrito en un lenguaje comprensible. El tiempo medio para aplicar el cuestionario sin conocimiento previo fue de 4 minutos y 45 segundos. Para la evaluación de la consistencia interna del instrumento, se ha utilizado la fórmula *Kuder Richardson* 20. La consistencia interna es de 0,71. El índice de concordancia del test Kappa se encuentra entre 0,983 y 0,797 para los distintos ítems.

**CONCLUSIONES:**

En este estudio se ha demostrado la equivalencia de contenido con el original. Además, la validación de las propiedades psicométricas establece que SPICT-ES^TM^ mantiene una adecuada fiabilidad y estabilidad. Si añadimos la satisfacción mostrada por los profesionales y la facilidad de uso, SPICT-ES^TM^ es una herramienta adecuada para la identificación de pacientes paliativos con enfermedades crónicas y necesidad de cuidados paliativos.

## INTRODUCTION

One of the difficulties faced by professionals working with chronic patients is the identification of which patients have palliative care needs. To date, at the international level, there are several questionnaires that have been designed to detect palliative patients, among them the RADboud indicators for Palliative Care Needs, RADPAC^1^, or the Prognostic Indicator Guide, GSF/PIG^2^. In Spain, the NECPAL CCOMS-ICO® questionnaire was also prepared by Gómez-Batiste et al.^3^

The fundamental objective of palliative care in any sanitary or socio-sanitary service is to alleviate suffering and improve as much as possible the quality of life of patients who have a chronic illness with a limited life prognosis^4^. In this sense, there are few objective identification criteria or validated tools to support physicians in the task of identifying a transition point to begin integrating palliative care^5^. For this reason, it is important to recognize the inflection point from which the patient will benefit from a more intense palliative care. The difficulty is to determine the moment that will mark the inclusion of this person in a specific palliative care. The establishment of this moment, marked by the separation between advanced chronic disease and palliative transition, has been defined by Boyd and Murray^6^. The importance of identifying it is based on avoiding the so-called “prognostic paralysis” of the professional, which can delay a change of focus in the patient for a long time. Professionals should be aware of the possibility that a patient can benefit from supportive and palliative care, fundamental for the offering of a better care at the end of his or her life^6^.

To this end, the University of Edinburgh Primary Palliative Care Research Group and the NHS Lothian (Edinburgh, Scotland) developed the SPICT^TM^ in 2010^7^, in order to create a guide that would identify and provide the necessary guidelines to carry out a plan of care in patients with palliative care needs. In this project, we used the version of the SPICT^TM^ questionnaire from November 2013, accessible at the following electronic address: http://www.spict.org.uk/the-spict/spict-es/spict-es-download/.

This article describes the process of translation, adaptation to Spanish, and validation of the psychometric properties of the SPICT^TM^, in order to establish the validity of content, reliability, and stability of the scale that will allow us to confirm that it is equivalent to the original. The SPICT^TM^ has 27 items with a yes/no dichotomous response and a final compartment in which the necessary plan of care is defined in case the patient is identified as needing palliative care. The 27 items are grouped into two categories: the first one with general indicators of health deterioration, and the second one with clinical indicators of advanced disease. The identification of the patient with palliative needs is based on the existence of at least two indicators of general health deterioration associated with an indicator of advanced disease.

The SPICT-ES^TM^ is a simple, one-page tool with indicators of health deterioration, usually present in advanced diseases, and a language easily understandable for its completion. Data show that this tool identifies 48% of all those who will die within the next 12 months after completing the SPICT criteria. This questionnaire has a sensitivity of 70% and a specificity of 87%, according to a study with 1,546 patients aged over 70 years in a health center^8^.

The purpose of this study was to translate, cross-culturally adapt to Spanish, and validate the Spanish version of the SPICT-ES^TM^ in order to comply with the criteria of equivalence with the original.

## METHODS

This project was developed as a multicenter and national research with the collaboration of the Galician Health Service, Health Service of the Balearic Islands, and the CUDECA Foundation (*Fundación de Cuidados del Cáncer*) of Malaga. The three organizations that participated have the necessary experience to carry out this project. The first two are responsible for coordinating the Plans and Strategies for Palliative Care in their Autonomous Communities. The third one develops an important work as an institution that provides comprehensive care to cancer patients and their families, from the creation of a complete program of palliative care, information, training, and research in the Malaga area.

In order to carry out this study, we first asked for the permissions needed from the authors of the original tool to adapt it to Spanish. At that time, a central committee of seven experts was set up to coordinate and review the documentation. Then, we followed the translation and back-translation method proposed by Beaton et al.^9^ For this, we established the following steps (Figure):

1. Translation: the Spanish translation of the original version was carried out by three independent experts fluent in English. The three translations sought to keep the equivalence of content with the original. Subsequently, a first consensus version (v1) was created between the three versions with a documented report on the different discrepancies and how they were solved.
2. Administration of the first version: this version was administered by a group of 22 professionals specialized in palliative care, establishing a proportional subsample between the three institutions in order to evaluate the difficulty of understanding the scale and the time required for the application. The results were analyzed by the central committee of experts to agree on a second version of the scale (v2).
3. Back-translation: the consensus version of the scale was translated into the original language in order to prove the equivalence of the content of the items of the scale with the original. Two back-translations were carried out by two independent bilingual translators who did not know each other in order to create two independent back-translations. One of the translators speaks Spanish as a native language and the other one English; both were unaware of the original questionnaire. The group of experts analyzed the back-translated versions along with the original to carry out a new consensus version (pre-final version) to maintain the semantic, idiomatic, and concept equivalence with the original.
4. Pilot test of the pre-final version: the pre-final version was evaluated in a pre-test by a group of 30 health professionals to evaluate the comprehensibility of the scale and to verify what the professional would estimate as the meaning of each item and answer.
5. Presentation of the documentation to the authors of the original questionnaire in order to prove that all steps were taken in the development of this adaptation of the SPICT^TM^ to Spanish.

**Figure:**
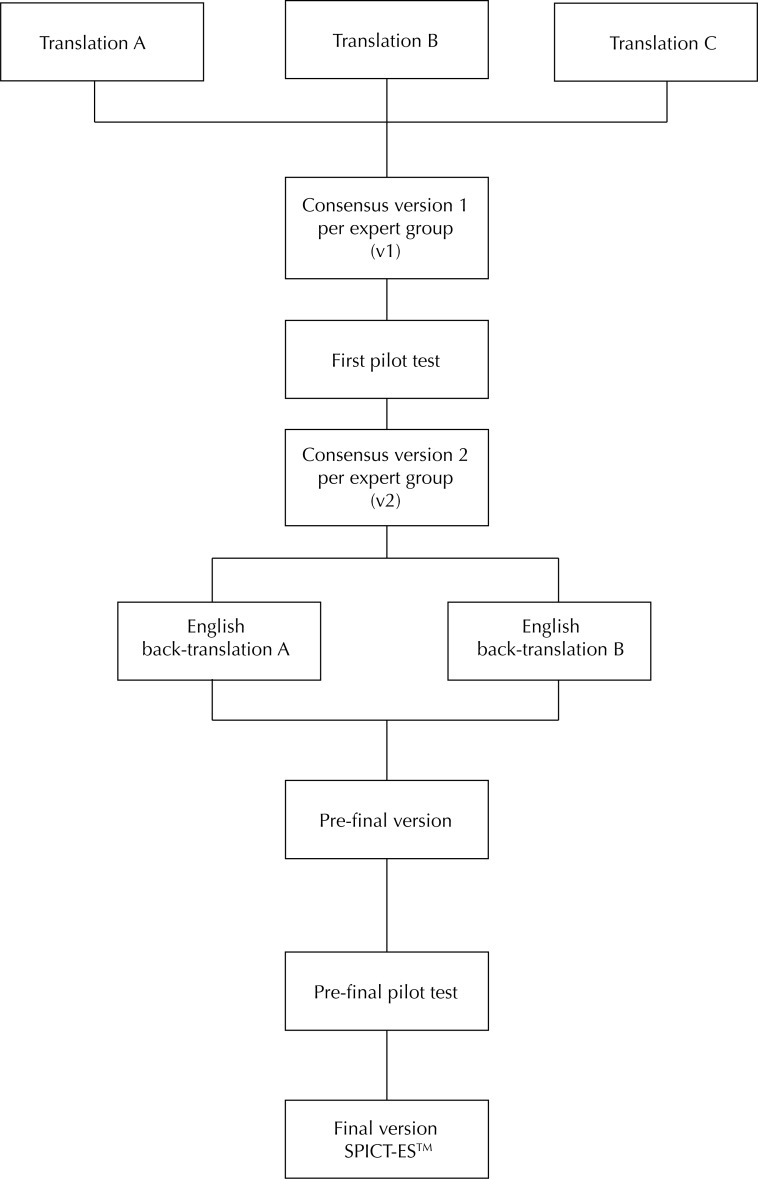
Scheme of the process of adaptation of the SPICT-ES^TM^.

To evaluate the comprehensibility of the tool, we used the debriefing methodology with health and non-health professionals^10^ ^,^ ^11^ to identify the words or concepts that were difficult to be interpreted, determine questions that cannot be answered with precision or that expose doubts in their answer, evaluate the sensitivity of the questions, and obtain suggestions for the reviewing of the wording of the questions in the questionnaire.

### Validation of the Psychometric Properties of the Scale

In a second phase, the psychometric properties of the Spanish version of the SPICT-ES^TM^ were evaluated, in order to know the internal consistency and stability of the tool. For this phase, and following the recommendation of Nunnally^12^, we determined that the minimum number of subjects required for the validation of the psychometric properties should be five times greater than the number of items. In this case, 135 patients would be needed as the questionnaire has 27 items. We established 30% for non-responses, so the minimum number defined as definitive was 176 patients.

### Statistical Analysis

To carry out the statistical analysis necessary for the validation of the psychometric properties of the questionnaire, the programs SPSS v.13 and EPIDAT were used. We used the Kuder-Richardson 20 to calculate internal consistency and the kappa test for the evaluation of the stability of the tool or agreement in the same observer, using the test and retest evaluation by the same researcher with a difference of ten days between them.

### Ethical Aspects

This study was approved by the Research Ethics Committee of A Corunna-Ferrol with the code 2014/563, on December 18, 2014.

## RESULTS

### Results of the Content Validity with the Original

During the procedure of adapting the tool to Spanish, the following detected discrepancies needed a special agreement among researchers:

1. The item “lives in a nursing care home or NHS continuing care unit, or needs care to remain at home” was translated to “institutionalized or needs care to remain at home”, as the network of hospitals of continuous care is scarce in Spain and, in addition, the concept of institutionalization already includes all the patients that are part of the health system.
2. The item *“*choosing to eat and drink less; difficulty maintaining nutrition” was translated as “decreased intake, difficulty maintaining adequate nutrition”. In this case, the concept of *choice* by a patient with dementia was debated by the group of experts, who finally opted for the concept of decreased intake. The concept of choice in a dependent patient did not have sufficient consistency if he or she had some form of dementia.

Subsequent administration of the first version was performed on a sample of 30 health professionals, and the average time to complete the questionnaire was 4 minutes and 45 seconds the first time it was applied by the professional.

For 95.5% of the participants, the questionnaire was simple to complete, and 90% considered it written in an understandable language. The main results of the administration phase are in Table 1.

**Table 1:** Results of the pilot phase.

Number of individuals according to general health deterioration	n (%)
Performance status is poor or deteriorating, with limited reversibility	18 (26.8)
Unplanned hospital admission on two or more occasions in the last six months	12 (17.9)
The person has had significant weight loss over the last 3 to 6 months or body mass index < 20	6 (8.9)
Persistent symptoms despite optimal treatment of underlying condition(s)	14 (20.9)
The person is institutionalized or needs care to remain at home	14 (20.8)
The person (or family) asks for palliative care; chooses to reduce, stop or not have treatment; or wishes to focus on quality of life.	3 (4.5)
Type of disease	n
Cancer	17
Dementia or frailty	21
Kidney disease	11
Neurological disease	7
Heart or vascular disease	9
Respiratory disease	8
Liver disease	2

### Result of the Validation of the Psychometric Properties

We obtained a final sample of 188 individuals. Mean age is 82.71 years (SD = 8.85). The age range of the patients is between 52 years and 101 years. The test-retest interval is 10.6 days. The main descriptive results of the validation phase are in Table 2.

**Table 2:** Results of the validation phase of the psychometric properties of the scale.

Variable		n	%
Total patients		188	100
Sex			
	Men	57	30.3
	Women	131	69.7
Autonomous community			
	Galicia	124	66.0
	Balearic Islands	64	34.0
Place of care			
	Primary Care Center	112	59.6
	Socio-sanitary residence	76	40.4
Disease indicators			
	Oncology	32	17.0
	Dementia/frailty	148	78.7
	Kidney disease	54	28.7
	Neurological disease	88	46.8
	Cardiovascular disease	90	47.9
	Respiratory disease	48	25.5
	Liver disease	11	5.9
Professional conducting the study			
	Physician		46.8
	Nurse		53.2

### Internal Consistency Analysis

In order to evaluate the internal consistency of the instrument, we used the Kuder-Richardson formula 20 (KR-20), since it is the most appropriate test for the scale based on dichotomous indexes. The result obtained for internal consistency is 0.71. This value allows us to affirm that it presents an adequate index of internal consistency since it is in the range between 0.70 and 0.90.

### Analysis of the Stability of the Scale

To evaluate the stability of the scale, we used the index of agreement of the Kappa test, with a test-retest analysis by the same observer. In the study design, we determined that it would be performed 10 days after the first one. The intraclass correlation coefficient data is in the range between 0.983 and 0.797.

## DISCUSSION

Before a questionnaire is applied to the general population, it must be validated in a sample of patients. The validation process of a tool has its own methodology, which includes a standardized procedure to evaluate both its reliability and validity. One of the main conclusions of the systematic review published by Maas et al.^5^ is that, although several tools to identify patients with palliative needs have been developed, none have been validated or widely used in Europe. Further collaboration in the international development, implementation, and evaluation of these tools is recommended. This is one of the objectives of this study.

The Spanish version of the SPICT-ES^TM^ (http://www.spict.org.uk/the-spict/spict-es/spict-é-download/), created originally in English, was translated and adapted culturally to Spanish according to the methodological steps stipulated in the literature. It was evaluated according to experts and analyzed by groups of professionals, keeping the necessary semantic, idiomatic, cultural, and content equivalence with the original instrument. It presents an excellent acceptability by the professionals who used it, both the physicians and nursing staff of the Palliative Care Units, as well as in the Primary Care Centers or Residential Centers where it was used. The questionnaire took 4 minutes to be completed the first time it was used without previous training, which allows us to assume that it will decrease according to practice. A fundamental aspect of any measuring tool is, on the one hand, the definition of the patients that it wants to identify in the best possible way and, on the other hand, the quick application and short time to be filled. In this sense, we demonstrated in this article that the SPICT-ES^TM^ meets both requirements.

Until today, the only tool available for the Spanish population to identify patients with palliative care needs is the NECPAL-CCOMS-ICO^©^. The most recent version of the NECPAL-CCOMS-ICO^©^ 3.0 from 2016 considered a reduction of the items in relation to the original. In this sense, we estimate that both this tool and the SPICT-ES^TM^ can be compatible, and more studies are needed to identify at which point of the palliative transitions^6^ they are. As for the SPICT-ES^TM^, it is a tool that presents an adequate content validity in relation to the original, and according to the palliative care professionals who have adapted it to Spanish, it correctly identifies patients with care needs with adequate reliability and validity.

One of the limitations of this study was to define the time that must elapse from the test to the retest. In this case, we opted for 10 days. It may seem low, but we needed to adjust this time since the average stay of patients in palliative units is approximately eight days. Thus, the initial suggestion was reduced from fifteen days to 10 days.

An important difference of the SPICT-ES^TM^ in relation to other tools is the exclusion of the surprise question from the questionnaire for the identification of palliative patients. We consider that the surprise question implies the inclusion of subjective criteria that can alter the application of the questionnaire. The inclusion of objective criteria when defining the situation of palliative need is one of the important parts of this tool. Nevertheless, the surprise question and the SPICT-ES^TM^ are not incompatible. The surprise question is a general indicator, but it should not be used in isolation without a more extensive evaluation^13^, with the use of specific questionnaires and with a greater degree of objectivity that allow greater reliability.

The association of the SPICT together with another tool to evaluate the complexity of the palliative patient, such as the IDC-PAL^14^ questionnaire, would offer the best socio-sanitary resource for each person who is in a situation of advanced disease with a limited life expectancy. In addition to the obvious benefits for patients, it would be important to reduce the costs of health systems.

In this sense, several studies show that the application of unnecessary procedures to patients with a terminal illness produce an unjustified increase in health expenditure^15^. Bloom and Kissick^16^ have found that the cost of hospital care in the last two weeks of life in patients with palliative care needs was 10.5 times higher when compared to home care. This increase in hospital costs was related to the application of diagnostic and therapeutic procedures that were more technologically intensive, applied until the death of the patient. Similarly, Cheung et al.^17^, in a cohort study with 107,253 persons, have found that costs were 43% higher in cancer patients who received care according to an acute model compared to those who received non-aggressive care. In order to solve these problems, more tools are needed to improve the ability to identify and diagnose palliative patients. The final objective should be to improve the capacity for intervention at the end of life at the community level in order to change the current profile of care to a greater extra-hospital care of these patients^18^. We believe that the SPICT-ES^TM^ can be a useful tool for the quick identification of these patients because of its ease of use and reliability characteristics.

The SPICT-ES^TM^ is a new tool to identify patients with palliative needs in the Spanish-speaking population. It would be added to the NECPAL CCOMS-ICO© 3.0 for the same task. These questionnaires are fundamental for the establishment of identification criteria to be carried out in the early stages of a chronic disease to avoid being diagnosed with excessive anticipation or at a very late stage, which is the most frequent case, depriving patients of the benefits of receiving quality palliative care. In the future, new studies should be carried out to identify the specificity and sensitivity values of the tool more accurately in a larger sample. There is also the need to integrate it into the evaluation of the patient as an advanced chronic disease, with personalized evaluation plans for each patient.
